# Non‐Contact Transspatial Regulatory Function of the Extracellular Matrix

**DOI:** 10.1002/smmd.70046

**Published:** 2026-07-24

**Authors:** Ting Cao, Chen Yang, Zhengyang Wang, Changmin Shao, Ziye Xu, Baode Chen, Jiayu Zhang, Yongcheng Wang, Fangfu Ye

**Affiliations:** ^1^ Department of Laboratory Medicine of the First Affiliated Hospital & Liangzhu Laboratory Zhejiang University School of Medicine Hangzhou China; ^2^ Zhejiang Key Laboratory of Clinical In Vitro Diagnostic Techniques Hangzhou China; ^3^ Beijing National Laboratory for Condensed Matter Physics and Laboratory of Soft Matter Physics Institute of Physics, Chinese Academy of Sciences Beijing China; ^4^ Oujiang Laboratory (Zhejiang Lab for Regenerative Medicine, Vision and Brain Health) Wenzhou Institute University of Chinese Academy of Sciences Wenzhou China; ^5^ School of Traditional Chinese Medicine Shandong Medical and Pharmaceutical University Yantai China

**Keywords:** extracellular matrix, macropinocytosis, non‐contact, remote regulatory

## Abstract

As a crucial biological material, the extracellular matrix (ECM) constitutes a major part of the extracellular microenvironment, offering both structural support and essential biological signals to surrounding cells. Research into ECM properties not only deepens our understanding of cellular behavior and metabolism but also opens new avenues for studying disease mechanisms and therapies. In this work, we report a previously unrecognized function of ECM by leveraging a three‐dimensional spatial cavity structure. It is termed “Non‐contact Transspatial Regulatory” (nCTR), referring to the ECM that remotely regulates cells without traditional direct physical contact. Systematic investigation and validation of key factors reveal that nCTR strongly depends on the 3D spatial distance between ECM and cells, governed by both physical spatial confinement and chemical signaling exchange. The process involves multiple components, pathways, and mechanisms, with macropinocytosis playing a dominant role. This newly identified function of the ECM offers a fresh perspective on cellular behavior and will undoubtedly open a new frontier in the cellular microenvironment studies.

## Highlights

- A custom‐designed 3D study model utilizing two biomaterials with reverse thermal properties is developed to investigate non‐physical contact interactions between the extracellular matrix (ECM) and cells.
- The Non‐Contact Transspatial Regulation (nCTR) function of the ECM is introduced for the first time, defined as the ECM's capacity to remotely modulate cell behaviors across three‐dimensional space without requiring direct physical contact.
- This regulatory function demonstrates strong spatial dependence on the distance between the ECM and target cells, and is jointly constrained by physical barriers and chemical exchanges.
- Mechanism analysis reveals that nCTR engages multiple signaling pathways and molecular components, with macropinocytosis serving as a potential mediator.
- The proposed nCTR will broaden the traditional framework of cellular microenvironment research and open promising avenues for advancing ECM biology.

## Graphical abstract

Non‐contact transspatial regulation of the extracellular matrix (ECM), whereby the ECM remotely modulates cellular behavior without direct physical contact, is proposed and systematically investigated for the first time. This process involves multiple pathways and components, with actin‐dependent macropinocytosis playing a predominant role potentially. This previously unrecognized property of the ECM expands the conceptual scope of cellular microenvironment research and opens new avenues for ECM‐related biology.

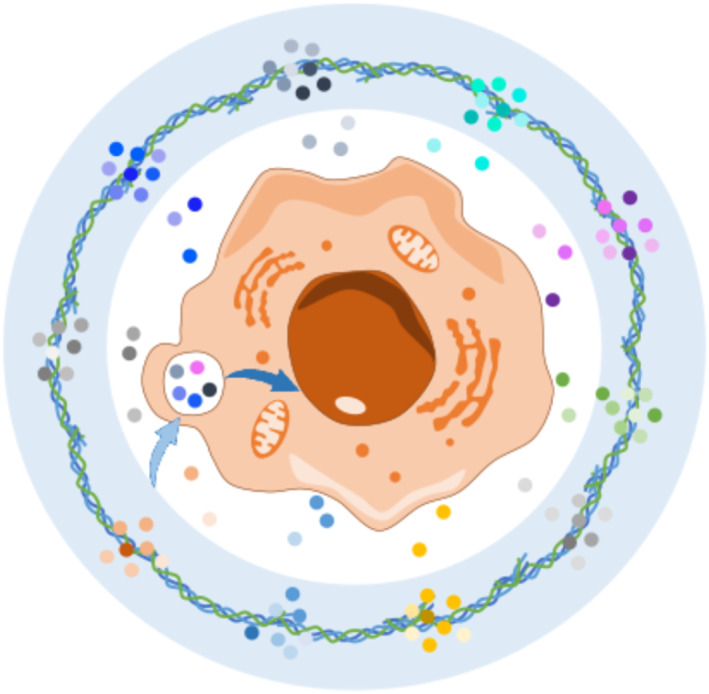

## 1 Introduction

The extracellular matrix (ECM) is a dynamic three‐dimensional (3D) meshwork consisting of reciprocally interwoven proteins and glycans. It represents all non‐cellular components of tissues that provide physical support and drive biological signaling for surrounding cells [1, 2]. In humans, the ECM primarily exists in two forms, namely interstitial ECM with fibrous meshwork and basement membrane with sheet‐like networks [2]. Generally, the ECM communicates with cells through direct donor‐receptor binding interaction for conveying physical, chemical, and biological cues. For instance, integrins, the principal transmembrane receptors embedded in the plasma membrane, mediate ECM‐cell interaction by linking ECM scaffold proteins, such as collagen fibers, fibrins, and laminins, to cytoskeletal proteins [3]. Beyond direct binding, the ECM may also confine cells through mechanosensitive ion channels in response to mechanical force [4, 5], but it is elusive due to the crosstalk between integrin mediated signaling and ion channel mediated pathways [3]. In short, the ECM plays a fundamental role in modulating cellular behavior. It supports bioactive molecules and stroma cells and promotes organ regeneration and tissue damage repair [6, 7].

Deciphering ECM properties and functions is always a vital topic for the studies of cell microenvironment. The ECM mediates cellular activities, such as adhesion, movement, migration and invasion [8]. Concurrently, it participates in the activation of alternative signaling cascade pathways for cellular survival, proliferation, differentiation, and gene expression [9]. Previous studies from our group have shown that ECM facilitates cell migration through matrix‐transmitted long‐range mechanical forces [10], enhances macrophages targeting toward cancer cells [11], and assists precise neuronal connectivity [12]. A well‐established mechanism underlying these phenomena involves direct binding between ECM collagen fibers and cellular integrins, leading to matrix remodeling dynamically through cell‐mediated contraction. The regulation mainly depends on physical connectivity between ECM constituents and cell surface receptors. Nevertheless, given the multifaceted roles of the ECM, it remains unclear whether non‐contact interactions (acting in the absence of direct molecular binding between ECM skeleton and cells) also contribute to its regulatory capacity. Elucidating this potential mode of ECM‐cell communication is critical for a comprehensive understanding of its functions in cellular microenvironment studies.

Recently, we have identified a previously uncharacterized mode of ECM‐mediated regulation, where the ECM modulates cellular behavior in a manner independent of conventional direct physical binding. It is termed as a non‐contact transspatial regulatory (nCTR) function of the ECM. Using a custom‐designed 3D spatial cavity model, we demonstrate that nCTR is a broadly conserved phenomenon modulated by both spatial distance and molecular exchange between the ECM and cells. Transcriptomic profiling reveals that nCTR is potentially mediated by macropinocytosis, facilitating the transfer of ions, proteins, and matrix vesicles. The deduction is then further validated by cellular inhibition assays. Collectively, this work uncovers a novel mode of ECM‐cell communication that fundamentally expands the current understanding of ECM biology and the tumor microenvironment. These findings provide critical insights into the mechanisms underlying disease pathogenesis, with significant implications for tumor initiation, progression, and recurrence.

## 2 Results and Discussion

### 2.1 Non‐Contact Transspatial Regulatory (nCTR) Function of the ECM

**FIGURE 1:**
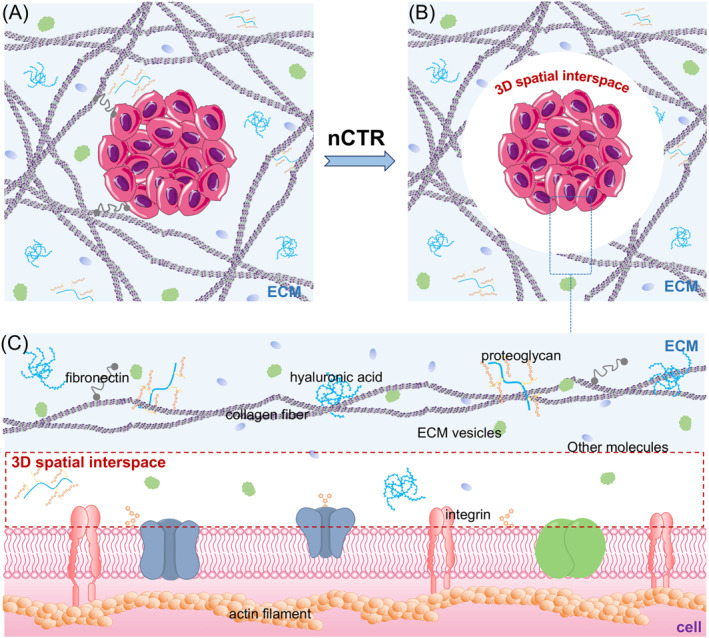
Illustration of the nCTR function of the ECM. (A) Traditional cognition of interaction between ECM and cells: components in ECM bind to cells by cell surface receptors, such as integrins. (B) A 3D spatial interspace structure is formed to study the nCTR function of ECM on cells. (C) The partial enlarged detail for separated spatial structure: ECM, 3D spatial interspace, and cells.

As a common biomaterial in human tissues, the ECM modulates cellular behaviors and metabolism through both biochemical and biophysical signals. These regulatory effects are primarily mediated via interactions between ECM components and specific cell surface receptors (Figure 1A). For instance, the ECM regulates anchorage‐dependent functions of epithelial cells via integrins [13], and its stiffness affects polarization and function of macrophages through non‐selective Ca^2+^‐permeable channel receptor Piezo1 or non‐selective transient receptor potential (TRP) cation ion channels [14]. Interestingly, emerging evidence suggests that the ECM can modulate cellular activities even in the absence of direct physical contact. As illustrated in Figure 1B,C, a 3D spatial gap physically separates the ECM from the cells, thereby precluding traditional ligand‐receptor interaction between the ECM skelemin and cells. Under this situation, remote ECMs are still capable of influencing cell morphology and dynamics, with some cells exhibiting behavioral similarities to those in direct contact with the collagen matrix. This observation implies the existence of a previously unrecognized form of ECM–cell communication, which we term the nCTR function of the ECM.

### 2.2 Cavity Structure Formation Characterization

**FIGURE 2:**
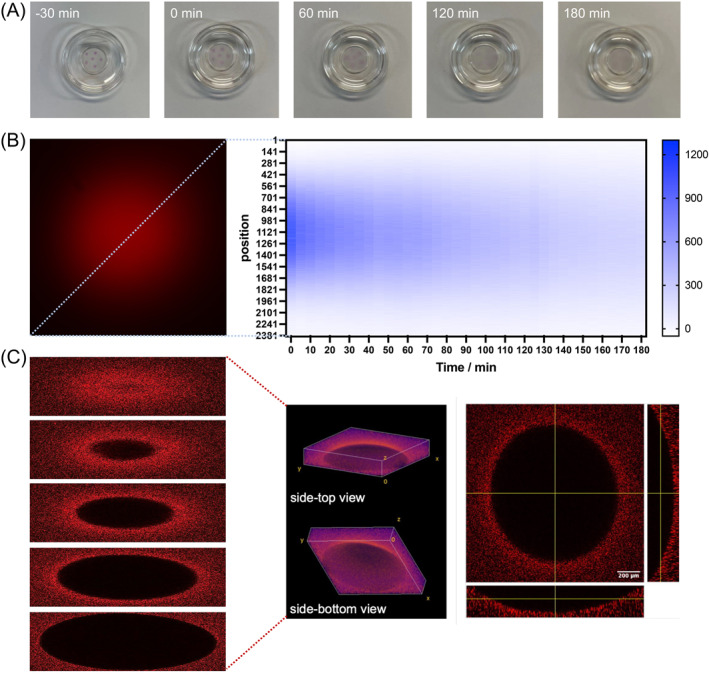
The characterization of the custom‐designed 3D spatial cavity compartment. (A) The record of diffusion of DMEM and gelatin at different time points; (B) the fluorescent signal record of rhodamine‐gelatin mixture in cavity structure; (C) the image and 3D reconstruction of the cavity structure by fluorescent beads‐embedded collagen gel.

The 3D spatial cavity compartment is created beneath a collagen gel through a solidification‐diffusion process, leveraging the inverse thermo‐responsive behavior of gelatin and collagen at different temperatures. To characterize cavity formation, the diffusion of 5% gelatin, prepared by mixing 10% gelatin with an equal volume of DMEM, was initially monitored (Figure 2A). Due to the presence of phenol red in DMEM, the mixture is visually distinguishable at the initial time point (−30 min). At 37°C, gelatin undergoes a gel‐to‐sol transition (liquefaction) and gradually melts; simultaneously, the collagen precursor undergoes fibrillogenesis and completes a sol‐to‐gel transition (gelation) (0 min). Over the next 1 h, phenol red in DMEM diffuses continuously, leading to a further expansion and faint visibility of red spots (60 min). At 120 min and 180 min, the diffusion process is almost completed, as indicated by the near disappearance of the red spots. This process was further corroborated by tracking the fluorescence of rhodamine‐gelatin loaded into the cavity (Figure 2B). With a molecular weight approximately seven times of gelatin, rhodamine reaches near‐complete diffusion at 90 min. A duration of 180 min is therefore confirmed as sufficient for full rhodamine diffusion and for minimizing potential interference from residual gelatin on cellular behavior.

The formed cavity structure can be visualized using reflective‐mode confocal microscopy to reconstruct the collagen fiber network (Supporting Information S1: Figure S1). To better characterize the cavity architecture, collagen gels embedded with fluorescent beads are imaged and three‐dimensionally reconstructed by Fiji software [15]. As shown in Figure 2C, the gel morphology is clearly rendered based on the bead‐derived signals. The cavity volume was estimated using the formula for half an ellipsoid:

V=12·43πabc=12·43π1500μm2·1400μm2·230μm=0.253μL

This calculated volume is close to the initially injected volume (0.25 μL), ignoring deviations from an ideal geometric shape. Theoretically, in the absence of direct ECM‐receptor (particularly integrin) interactions, cells in the cavity would be expected to exhibit phenotypes similar to those under conventional dish culture, given that the cavity is more than 10 times larger than the cells. Surprisingly, we observed that cells displayed distinct morphological and behavioral dynamics relative to standard dish cultures, implying the presence of other factors in cell behavior regulation.

### 2.3 Cell Morphology Characterization

**FIGURE 3:**
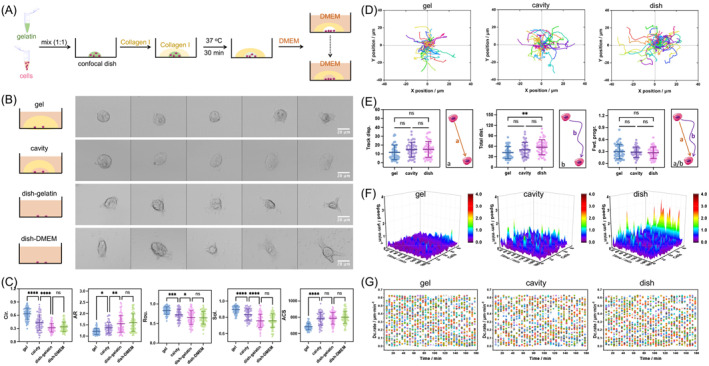
The influence of nCTR on cell morphology and dynamics. (A) Simplified flow diagram of the formation of cells‐in‐cavity structure underneath hydrogel; (B) the cellular morphologies of gel, cavity, dish‐gelatin and dish‐DMEM samples. Dish‐gelatin means cell‐gelatin mixture is placed on dish to culture, while dish‐DMEM means cell suspension in DMEM is placed on dish to culture; (C) quantification of cell shape descriptors between different samples. Trajectory analysis of cells in gel, cavity, and dish samples: (D) Cell movement trajectory recorded with normalized starting point for cells; (E) the calculation of *Track disp*., *Total dist*., and *Fwd*. *progr*. of cells; (F) kinematic velocity statistics during the tracking process; (G) the distribution of *Dc*. *rate* for cells under different microenvironments.

To quantitatively assess the non‐contact regulatory effects (nCTR), we first analyze cell morphology using multiple parametric descriptors. As illustrated in Figure 3A, a cell‐in‐cavity structure is formed based on the inverse thermo‐property of gelatin and collagen. Briefly, the cell‐gelatin mixture is solidified on a confocal dish at 4°C, followed by the coverage of a collagen precursor solution. Subsequent incubation at 37°C induces collagen gelation while triggering gelatin liquefaction and diffusion out of the collagen network, ultimately generating a hollow cavity beneath the collagen gel containing the cells. Microscopic examination revealed distinct morphological differences across conditions (Supporting Information S1: Figure S2). A majority of cells directly embedded under collagen gel (designated as the gel group) exhibit a rounded morphology (Figure 3B), consistent with physical confinement imposed by the surrounding matrix [3]. In contrast, cells cultured on conventional dishes (labeled as dish‐gelatin and dish‐DMEM groups) displayed extensive adhesion and spreading, accompanied by prominent lamellipodia formation (Figure 3B). Notably, cells within the cavity space underneath the hydrogel (referred to as the cavity group) exhibit an intermediate phenotype: a subset of cells retains a rounded conformation, while others adopt a spread morphology.

To systematically evaluate phenotypic differences among cells under different culture conditions, we quantify morphological differences using five geometric descriptors: circularity (Cir.), aspect ratio (AR), roundness (Rou.), solidity (Sol.), and average centroid size (ACS). These parameters were computed according to the following mathematical expressions:

(1) Circularity(Cir.)=4π·Areaperimeter2

(2) Aspectratio(AR)=MajorAxisMinorAxis

(3) Roundness(Rou.)=4·Areaπ·Majoraxis2

(4) Solidity(Sol.)=AreaConvexarea

(5) $$\text{Average}\;\text{Centroid}\;\text{Size}\;\left(\text{ACS}\right)=\sqrt{\frac{1}{N}\;\sum_{i=1}^{n}d_{i}^{2}}$$

*d*_*i*_ in Equation (5) means the distance of the point on the outline from the centroid of the shape.

Single cells within the cavity, without direct contact with the collagen gel, are identified (Supporting Information S1: Figure S2) and their morphological parameters are statistically analyzed as shown in Figure 3C. To determine an appropriate data processing approach, two methods for handling outliers are evaluated: outlier deletion (all data from a cell are excluded if any of its parameters is classified as an outlier) and outlier deficiency (only the specific outlier parameter is removed). The results summarized in Supporting Information S1: Table S1 demonstrate that both methods yield datasets with consistent trends. Given the advantage of retaining more raw data, the outlier deficiency method was selected for all subsequent analyses.

The distributions of morphological parameters across the four experimental groups are presented in Supporting Information S1: Figure S3, with almost all data presenting a normal distribution tendency. We first compare shape descriptors related to cell spreading and morphology under conditions with or without gelatin. The absence of significant differences between the dish‐gelatin and dish‐DMEM groups (Figure 3C and Supporting Information S1: Figure S4) indicates that gelatin itself has negligible effects on cell morphology under present conditions. Therefore, the dish‐gelatin was designated as the control experiment (referred to the dish group) for subsequent experiments. Notably, as shown in Figure 3C, the values of Cir., AR, Rou., Sol. and ACS for cells in the cavity consistently fall between those of the gel and dish groups. This intermediate phenotype suggests that a subset of cells in the cavity is influenced by the collagen hydrogel despite the absence of physical contact. This result, with our custom‐designed experimental model, provides the first experimental evidence for the existence of nCTR.

### 2.4 Cell Dynamic Tracking

To investigate the role of nCTR in cell migration, we have performed cell dynamic tracking for gel, cavity, and dish samples (Supporting Information S1: Figure S5). As illustrated in Figure 3D, cells under the hydrogel exhibit restricted mobility. Their adhesion to the collagen network imposes spatial constraints, resulting on average in sluggish and short‐range movement. In contrast, cells cultured on the dish surface displayed faster and more extensive migration. A well‐spread morphology appears to facilitate motility, enabling cells to explore broader regions through combined forward, backward, and turnaround movements. Notably, cells localized within the cavity again exhibit an intermediate phenotype: some cells move over relatively long distances while others move locally. We quantitatively evaluate migratory behavior using three metrics by Trackmate [16] (Figure 3E): Track displacement (Track disp.), total distance traveled (Total dist.) and linearity of forward progression (Fwd. progr.). Although no significant difference between adjacent data pairs is observed, the data still show a tendency that cells in the cavity group display an intermediate *Track disp*., *Total dist*. and *Fwd*. *progr*. value, consistent with the phenomenon in cell morphology analysis. Interestingly, the result of *Fwd*. *progr*. exhibits an inverse trend (0.298 for the gel group, 0.281 for the cavity group, and 0.268 for the dish group), implying that confined cells may migrate more directionally compared with unconfined counterparts. We further analyze the raw instantaneous kinematic velocity over the entire tracking period. The kinematic velocity exhibits a gradual increasing trend from the gel group, cavity group, to dish group (Figure 3F). The average migration speeds are 0.23 μm/min (gel), 0.28 μm/min (cavity), and 0.32 μm/min (dish), respectively. Notably, the mean directional change rate (*Dc*. *rate*) remains nearly identical across the three groups (Figure 3G), with values of 0.29, 0.30, and 0.29 μm/min for the gel, cavity, and dish groups, respectively. These observations indicate that cells under different microenvironmental conditions undergo adaptive changes in their motility. These findings further support the existence of a non‐contact regulatory mechanism.

### 2.5 Key Factors Exploration

**FIGURE 4:**
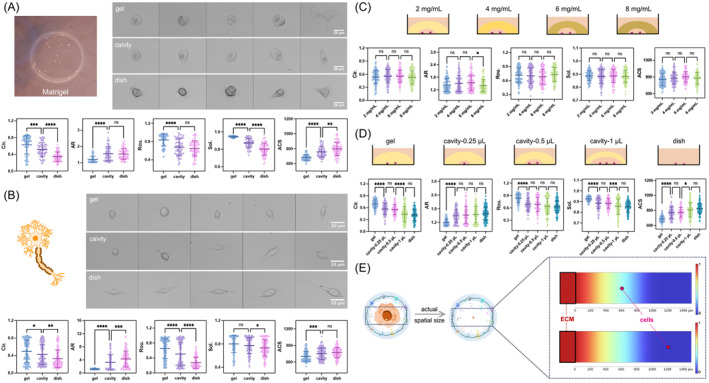
The exploration of cell type, ECM types, collagen concentration, and ECM‐cell distance for the nCTR function. (A) The investigation of cell status with Matrigel: cellular morphology record and quantification of the shape descriptor parameters; (B) the investigation of cell status with PC12 cells: cellular morphology record and quantification of the shape descriptor parameters; the influence of (C) ECM concentration and (D) the distance between ECM and cells on cell morphological phenotype; (E) COMSOL simulation analysis for the remote interaction between ECM and cells (the distance between ECM and cells is far away from the radius of the cells).

To identify key factors underlying nCTR, we have investigated the behaviors of MDA‐MB‐231‐GFP cells embedded in a Matrigel cavity and PC12 neuronal cells within a collagen cavity. Matrigel, sourced from Engelbreth–Holm–Swarm mouse sarcoma, is widely employed for 3D extracellular matrix reconstruction in organoid models [17]. Its composition includes diverse polysaccharides (e.g., hyaluronic acid and heparan sulfate), structural proteins (e.g., collagen IV and laminin), and numerous growth factors, representing a matrix system biochemically and structurally distinct from collagen hydrogel. As expected, MDA‐MB‐231 cells within the Matrigel cavity exhibit morphological trends consistent with those observed in prior collagen‐based experiments (Figure 4A). Representative images reveal a mixed phenotype: a subset of cells maintain a rounded morphology (similar to cells under Matrigel), while others adopt a spread morphology (similar to cells on dish). Quantitative analysis of shape descriptors further confirms the presence of nCTR‐like effects in the Matrigel system (Figure 4A). Similar regulatory pattern is also observed with PC12 cells in collagen hydrogel (Figure 4B). PC12 cells, derived from rat pheochromocytoma and widely used in neurobiological studies [18], display heterogeneous morphology across conditions. Microscopic evaluation (Figure 4B) shows that the extent of neurite outgrowth increases progressively from cells under gel to cells in cavity to cells on dish. Corresponding shape descriptor quantification confirms that cavity‐housed PC12 cells display an intermediate morphological state between gel and dish conditions (Figure 4B), further supporting the universality of nCTR across cell types and matrix compositions.

We further investigate the impact of ECM physicochemical properties on cellular responses, given their critical role in modulating cell behavior [3]. Firstly, the ECM concentration (varying from 2, 4, 6 to 8 mg/mL) was changed and the result is displayed in Figure 4C. Interestingly, morphological parameters exhibit no obvious correlation with the matrix concentration, suggesting 2 mg/mL is a sufficient concentration for the ECM's remote regulation on cells. We next evaluated how the spatial distance between the ECM and cells modulates morphological outcomes. As illustrated in Figure 4D, with the increase of the cavity volume, cellular morphology shows a shift tendency from a cell‐under‐gel phenotype toward a cell‐on‐dish phenotype. Cells in 1 μL cavities exhibit morphological characteristics nearly identical to those cultured on a dish, suggesting a loss of ECM‐mediated influence beyond a specific spatial threshold. As the component of the commercial collagen is relatively plain, the gap between the collagen hydrogel and the cells contains no collagen‐derived substances theoretically. Therefore, a possible mechanism for the nCTR is the physical spatial constraint imposed by the remote collagen gel. As illustrated in Figure 4E (a schematic simulation by COMSOL [19] simulation), the physical barrier of the ECM could restrict the inward diffusion of external chemicals from surroundings and the outward diffusion of autocrine or paracrine factors secreted by the cells, thereby forming a locally concentration‐gradient microenvironment for cell recognition within the cavity.

### 2.6 Transcriptome Analysis

**FIGURE 5:**
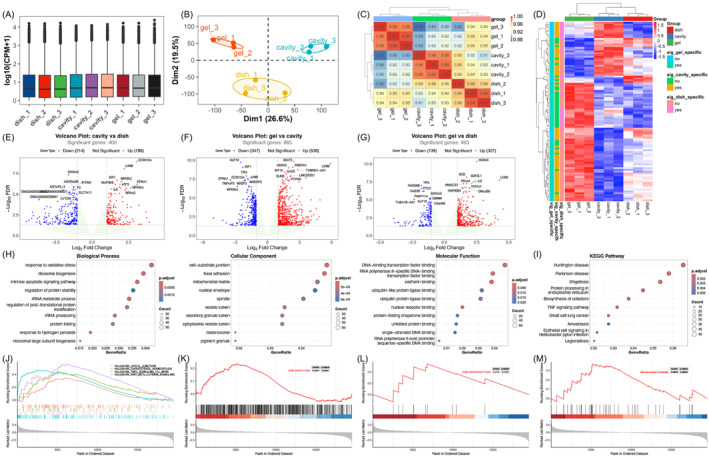
Transcriptome analysis of different experimental conditions. (A) The total gene expression, (B) PCA clustering, (C) Pearson correlation coefficients difference, and (D) top 100 DEGs in gel, cavity, and dish samples; the volcano plot of the DEGs between (E) cavity VS dish, (F) gel VS cavity, and (G) gel VS dish samples; the top 10 terms of (H) GO and (I) KEGG pathway enrichment for the DEGs between cavity and dish samples; (J) the four enriched gene sets involving substance exchange process for GSEA analysis; The result of enrichment in (K) GOBP_ENDOCYTOSIS gene set, (L) GOBP_MACROPINOCYTOSIS gene set, and (M) self‐established macropinocytosis‐related gene set MACROPINOCYTOSIS _complete65.

Transcriptome analysis was performed using three valid biological replicates per group to identify cellular gene expression potentially influenced by nCTR (Supporting Information S1: Figure S6). Overall, the three groups exhibited similar global gene expression levels (Figure 5A) but distinct expression profiles (Figure 5B). Cavity samples show a higher Pearson correlation with dish samples than with gel samples (Figure 5C), supporting the hypothesis that cells in the cavity experience a culture microenvironment comparable to that of dish‐cultured cells. Differential expression gene (DEG) analysis, performed by edgeR [20], also shows a closer gene expression between the cavity and dish groups (Figure 5D). Pairwise DEG comparisons were visualized using volcano plots (Figure 5E–G). Between cavity and dish groups (Figure 5E), the cavity condition is associated with classical type I collagen‐based matrix remodeling, marked by such as the upregulation of *COL1A1*. In contrast, the gel condition exhibited an obvious change in gene expression relative to the cavity condition, with 885 DEGs and logFC values ranging from approximately −5.0 to +5.9 (Figure 5F). Gel‐cultured cells showed enhanced extracellular matrix production and remodeling, evidenced by upregulation of *COL1A1*, *COL5A1*, *COL6A3*, and *COL15A1*, consistent with a scaffold‐like microenvironment. The comparison between gel and dish groups (Figure 5G) represent fundamentally distinct cellular states, with the upregulation of *COL1A1*, *COL5A1*, *COL27A1*, *FN1*, *LAMB2*, *DDR2*, *LTBP2*, *JUP*, *CLDN1*, *CLDN7*, and so on.

We then performed Gene Ontology (GO) [21] and Kyoto Encyclopedia of Genes and Genomes (KEGG) [22] enrichment analyses using DEGs identified between the cavity and dish groups. A total of 108 biological process (BP) terms, 71 cellular component (CC) terms, 14 molecular function (MF) terms, and 34 KEGG pathways were enriched using default thresholds of *p*‐value cutoff < 0.05 and q‐value cutoff < 0.2 (Supporting Information S1: Tables S2 and S3). The enrichment results reveal that the transcriptional changes are predominantly centered on three interconnected biological themes: proteostasis and endoplasmic reticulum (ER) stress, cellular stress responses, and inflammatory signaling. Specifically, the top enriched pathways (Figure 5H,I) include protein processing in the ER, ribosome biogenesis, rRNA metabolism, ubiquitin‐mediated proteolysis, oxidative stress, protein folding, and activation of the TNF signaling pathway. These results collectively point to material exchange processes involving intracellular trafficking, protein transport, and membrane compartment dynamics. GO analysis shows significant enrichment of terms associated with late endosomes (GO:0005770), lysosomal membranes (GO:0005765), and vesicle lumens (GO:0031983), key hubs in the vesicular trafficking network. Complementing these observations, KEGG analysis highlights robust enrichment of pathways involving material exchange, including protein processing in the ER (hsa04141), protein export (hsa03060), ubiquitin‐mediated proteolysis (hsa04120), proteasome pathways (hsa03050), and so on.

To further investigate the mechanism underlying nCTR, we have performed Gene Set Enrichment Analysis (GSEA) [23] using all DEGs identified between the cavity and dish groups. A total of 12 hallmark gene sets is enriched (Supporting Information S1: Figure S7), four of which are associated with substance exchange processes (Figure 5J). Consistent with this observation, the GOBP_ENDOCYTOSIS gene set showed significant enrichment (Figure 5K). More refined analysis revealed enrichment of gene sets related to specific endocytic routes, including GOBP_PINOCYTOSIS, GOBP_MACROPINOCYTOSIS, GOBP_CLATHRIN_DEPENDENT_ENDOCYTOSIS, and GOBP_CAVEOLIN_MEDIATED_ENDOCYTOSIS (Figure 5L and Supporting Information S1: Figure S8). Given the limited number of genes (11 genes) in the core macropinocytosis‐associated gene set, we have constructed an expanded macropinocytosis‐related gene set comprising 66 genes based on protein–protein interaction networks in the STRING database [24] (Supporting Information S1: Figure S9). Evaluation of this expanded gene set demonstrated increased edge enrichment, with a decreased *p*‐value of 0.089 (Figure 5M). These findings show a material exchange network at multiple levels, including secretory, endocytic, degradative, and nucleocytoplasmic pathways, with endocytic processes potentially playing a key role in the nCTR function of the ECM.

GO and KEGG enrichment analyses were also performed on the DEGs identified between the gel and cavity groups, with results presented in Supporting Information S1: Figure S10. These two groups exhibit significant enrichment in pathways associated with cell cycle progression, DNA replication, and chromosome dynamics, suggesting that the primary differences between them lie in cellular proliferation and metabolic activity. Consistent with this, GSEA revealed reverse enrichment of all related pathways (Supporting Information S1: Figure S11A–D). Notably, GOBP_PINOCYTOSIS, GOBP_MACROPINOCYTOSIS, and the self‐established macropinocytosis‐related gene set (Macropinocytosis_complete65) all show reverse enrichment, indicating that endocytic processes also potentially represent a distinguishing pathway for cells in the cavity condition relative to those in the gel condition. Analogous analyses were performed for DEGs between the gel and dish groups (Supporting Information S1: Figures S12 and S13). The gel condition exhibits a distinct gene expression profile characterized by enriched pathways related to DNA metabolism, protein folding, and stress response and is clathrin‐dependent compared with the dish condition.

### 2.7 Mechanism Exploration and Verification

**FIGURE 6:**
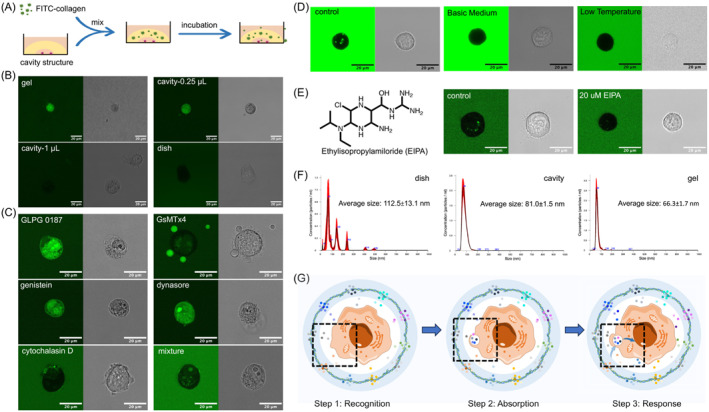
The inhibition reaction testing. (A) The diagrammatic drawing of the FITC‐collagen uptake detection into MDA‐MB‐231 cells; (B) the uptake result of FITC‐collagen in gel, cavity‐0.25 μL, cavity‐1 μL, and dish samples; (C) the inhibition effect of GLPG 0187, dynasore, genistein, cytochalasin D, GsMTx4, and the mixture of all five inhibitors on FITC‐collagen absorption for cavity‐0.25 μL sample; (D) the influence of the serum‐related pathways and low temperature on FITC‐collagen uptake; (E) the inhibition of EIPA for FITC‐collagen uptake; (F) the size distribution of obtained extracellular matrix vesicles for the dish, cavity, and gel samples; (G) a possible mechanism deduction for the realization of the nCTR function of ECM on cells.

To demonstrate the possible regulatory pathways of the ECM on cell expression, we employ FITC‐labeled collagen with a broad molecular weight distribution (0–25 kDa, Supporting Information S1: Figure S14) to simulate freely diffusible components derived from ECM or cells within the cellular microenvironment. As shown in Figure 6A,B, cells in the gel condition exhibit clear intracellular FITC‐collagen uptake, while those in the dish group show negligible fluorescence signal (the gel sample), indicating that uptake of FITC‐collagen is facilitated by the presence of ECM. Notably, cells in the 0.25 μL cavity group display uptake behavior similar to the gel group (the cavity‐0.25 μL sample), supporting the presence of ECM‐mediated regulation even in the absence of direct contact. In contrast, increasing the cavity volume to 1 μL suppresses the FITC‐collagen endocytosis, with most cells showing negligible fluorescent signal (the cavity‐1 μL sample). This distance‐dependent effect aligns with prior morphological observations (Figure 4D).

Next, we introduced some cellular inhibitors into the culture medium to assess their effects on cellular uptake of the FITC‐labeled collagen. Theoretically, collagen uptake by cells is generally mediated through two primary routes: receptor‐mediated active transport, typically involving integrin recognition, and endocytic pathways, including clathrin‐ or caveolin‐dependent endocytosis and nonspecific fluid‐phase macropinocytosis [25]. To systematically evaluate these possibilities, five common inhibitors involved in cell endocytosis were detected for the uptake of FITC‐collagen in cavity conditions (Figure 6C) after the evaluation of the safety concentration (Supporting Information S1: Figures S15 and S16).

GLPG 0187, a broad‐spectrum integrin receptor antagonist [26], does not significantly suppress FITC‐collagen uptake, suggesting that integrin‐mediated recognition is not the primary mechanism under present conditions. This is consistent with the physical separation between cells and the ECM and the way cells take up FITC‐collagen in our model. In contrast, both dynasore [27] (blocks the GTPase activity of dynamin and cell migration) and genistein [27] (a multiple tyrosine kinases inhibitor) show slight inhibition ability with decreased fluorescence signal. Cytochalasin D [27], an actin polymerization inhibitor inhibiting exosome release and endocytosis, shows the most effective inhibition ability with the obvious decreased fluorescent signal in cells. Together with the observed irregular fluorescent vesicles within cells, a possible speculation is proposed that macropinocytosis may serve as a possible route for FITC‐collagen internalization [28]. We also tested GsMTx4, a selective inhibitor of mechanosensitive ion channels such as Piezo and TRP [11]. While it does not directly target macropinocytosis, a subset of cells exhibits reduced uptake (Supporting Information S1: Figure S17), and residual fluorescent vesicles are detected in the system. These vesicles may represent ongoing internalization events or material derived from lysed neighboring cells, a phenomenon that occasionally occurred in other inhibitor treatments as well (Supporting Information S1: Figure S18). The mixture of all inhibitors resulted in a significant reduction of intracellular fluorescent signal (Figure 6C), confirming the collaborative role of multiple pathways for nCTR.

We further performed FITC‐collagen endocytosis assays under serum‐deprivation and energy‐dependent conditions, using shortened culture times. As shown in Figure 6D, cellular uptake of FITC‐collagen is largely inhibited under basal medium conditions (where growth factor pathways are not activated) or at reduced culture temperature (4°C, which limits energy availability for endocytic processes). More supportive evidence is obtained using EIPA (a well‐established inhibitor of macropinocytosis) (Figure 6E) [29] for the absorption test, in which cells show almost no fluorescent signal. Macropinocytosis leads to the non‐selective uptake of large amounts of extracellular fluid and proteins by cells. This abnormally enhanced influx of substances may impose an additional burden on the endosomal‐lysosomal system and the protein degradation system, thereby inducing protein homeostasis‐related responses such as ER stress and the unfolded protein response. This is in strong agreement with the GO and KEGG results of the transcriptome analysis.

Based on these findings, we propose the following potential mechanism underlying nCTR: the physical scaffold of the ECM provides spatial constraint that facilitates the mobility and cellular uptake of free‐moving substances within the cellular microenvironment, thereby enabling nCTR‐mediated signaling. This signaling influences multiple cellular activities, including morphology, motility, and even vesicular trafficking (Figure 6F). Thus, through the route of recognition‐absorption‐response (Figure 6G), ECM consequently realizes the remote regulation on cells.

## 3 Conclusion

In this study, we have established a 3D spatial interspace model leveraging the reverse thermosensitivity of gelatin and collagen. Within this system, a defined cavity is created inside the hydrogel, enabling investigation into the regulatory roles of the ECM on cellular functions. Utilizing this model, we have uncovered a previously unrecognized function of the ECM, termed nCTR. It describes the capacity of the ECM to regulate cells across a physically isolated 3D space.

To investigate the influence of nCTR on cell spreading and migration, we first characterize cellular morphology and dynamics under three different culture conditions: dish, cavity, and gel. Quantitative analysis of cell shape descriptors and motility parameters reveals that cells within the cavity exhibit an intermediate phenotype between those on the dish and those under gel, regardless of hydrogel biomaterials or cell types. Results also show that nCTR is attenuated with increasing spatial separation between the ECM and cells, whereas alterations in ECM concentration have minimal impact under the established experimental conditions. This spatial dependence implies that nCTR may function as a physical spatial constraint with respect to cellular sensing. Transcriptomic profiling identifies numerous up‐regulated DEGs and multiple biological processes for nCTR of the ECM. Further inhibitor assays indicate that macropinocytosis of freely diffusing components probably serves as a primary cellular mechanism facilitating nCTR in the aspect of chemical substance exchange.

The published remote regulation of cells by the ECM (e.g., soluble‐factor‐mediated signaling [30], cellular mechanotransduction [31], extracellular vesicle communication [32], etc.) may overlap with nCTR function, given the complexity of cargos mimicked by FITC‐collagen in this work. In this work, we highlight the newly identified role of the nCTR function of the ECM and propose that its function may be mediated through macropinocytosis. Further studies are needed to identify the active constituents and related cellular pathways. Additionally, since all experiments are currently based on in vitro cavity models, it remains unclear whether similar non‐contact ECM regulation occurs in vivo under physiological conditions.

In summary, the ECM can regulate cells in the absence of traditional physical contact. By employing a custom‐designed 3D spatial interspace model, we have demonstrated its prevalence and systematically investigated its characteristics. The regulatory exhibits a strong dependence on the spatial distance between the ECM and cells, and is primarily facilitated by macropinocytosis with a great potential. As a foundational concept, nCTR introduces a new view in ECM biology and offers fresh perspectives for understanding cellular response, which undoubtedly will open a new frontier in the cellular microenvironment studies.

## 4 Experimental Section

### 4.1 Cell Culture

MDA‐MB‐231‐GFP (Cat. 1101HUM‐PUMC000407) and MDA‐MB‐231 (Cat. 1101HUM‐PUMC000014) cell lines were obtained from Chinese Infrastructure of Cell Line Resource (Beijing, China) and cultured in DMEM medium (Gibco, Cat. 11965092), supplemented with 10% FBS (Fetal Bovine Serum, Gibco, Cat. 10091‐148) and 1% P/S (Penicillin‐Streptomycin, Corning, Cat. 30‐002‐CI). PC‐12 cell line (Cat.1101RAT‐PUMC000024) was also purchased from Chinese Infrastructure of Cell Line Resource and cultured in RPMI‐1640 medium (Gibco, Cat. 11875093), supplemented with 10% FBS and 1% P/S. All cells were normally incubated under a humidified atmosphere at 37°C and 5% CO_2_ (Thermo Fisher, USA), and passaged every 2–3 days after reaching about 80% confluency.

### 4.2 Cavity Formation

Generally, 10% (w/v, g/mL) gelatin (Aladdin, Cat. G274269‐100 g) was prewarmed in a 37°C water bath to convert the solid gel into liquid status, meanwhile 1.5 mL tubes were precooled at 4°C for collagen solution preparation before the experiment. MDA‐MB‐231‐GFP cells were washed twice with 1x PBS (Gibco, Cat. 10010023), then 2 mL 0.0125% Trypsin (Corning, Cat. 25‐050‐CI) was added into the 5‐mL cell culture flask. Cells were digested at 37°C for 3 min and then 3 mL DMEM complete medium was added to terminate the enzymolysis process. Cells were centrifuged at 100 g for 5 min. During this time, pre‐mixed Collagen I preparation solution was prepared according to the manufacturer's instructions: 10x PBS (Gibco, USA) and corresponding volume of 1 M NaOH (Sigma‐Aldrich, Cat. 28–301) were added into sterile water; high concentration Collagen I (rat tail high concentration, Corning, Cat. 354249) was added into the solution based on desired final collagen concentration; pH of the preparation solution was adjusted into neutrality or slightly alkalinity (pH Test strips 4.5‐10.0, Sigma‐Aldrich, Cat. P4536). After that, cells were collected and counted.

MDA‐MB‐231‐GFP cells with a final cell concentration of 4E5 cells/mL were prepared and mixed with prewarmed 10% gelatin (1:1, v/v) gently. Cell mixture with 0.25 μL was deposited onto confocal culture dishes (Cellvis, USA) with 10‐μL pipette tips (change pipette tips for each confocal dish to avoid mixture mucus) and cooled at 4°C for 12 min (estimated *t* = 12 + 3 · (V − 0.25)/0.25 min based on our experimental experience) to solidify the gelatin‐cell mixture. Premixed collagen solution with 200 μL was added to cover the gel aliquot and then the dish was put into 37°C incubator immediately for 30 min. After that, 1 mL fresh medium was added to the dish and incubated at 37°C for 1 h. The medium was replaced with new fresh medium each hour for three times. Then, the confocal dish was incubated at 37°C with no disturbance until used.

For the gel control experiment, 4E5 cells/mL MDA‐MB‐231‐GFP cells were mixed with fresh medium (1:1, v/v) and 0.25 μL cell suspension was deposited onto confocal dishes. The dish was cooled at 4°C for 12 min and then 200 μL premixed collagen solution was added to cover the cells. The following operations were the same as those in the cavity group.

For the dish control experiment, 4E5 cells/mL MDA‐MB‐231‐GFP cells were mixed with prewarmed 10% gelatin (1:1, v/v), and 0.25 μL gelatin‐cell mixture was deposited onto confocal dishes. It was cooled at 4°C for 12 min and then 200 μL culture medium was added. The dish was immediately transferred into a 37°C incubator for 30 min. The following operations were the same as those in the cavity group.

For MDA‐MB‐231 cells or PC 12 cells, the experimental operation was almost the same as MDA‐MB‐231‐GFP cells except that PRMI‐1640 medium was used for PC 12 cell culture.

For Matrigel formation, Matrigel solution (Corning, USA) was diluted with the same volume of 1x PBS, and then added into confocal dishes to cover gel aliquots. Dishes were incubated in 37°C incubator for 30 min to solidify Matrigel and the following experimental operations were the same as the cavity formation process.

The preparation of 10% Gelatin solution: 6 mL sterile water (Gibco, USA) was added into a 15‐mL tube; then, 1 g Gelatin was added and the tube was put in a 37°C water bath for one hour; water was added to 10 mL and the tube was placed in a 37°C water bath until a transparent and clear solution was formed. It can be stored at 4°C for at least 1 year.

### 4.3 Gelatin Dissolution Process

DMEM medium was mixed with prewarmed 10% gelatin and then deposited on a confocal dish. It was placed at 4°C for 12 min to gelatinize the solution. Then, 200 μL premixed collagen solution was added onto the dish to cover the gel aliquots and put into 37°C incubator immediately for 30 min. Sterile 1 mL PBS was added and the picture was recorded for the following 3 h, during which PBS was replaced with fresh PBS every 1 hour.

DMEM medium containing 10 μM 70 kDa rhodamine (Sigma‐Alrich, USA) was prepared and mixed with prewarmed 10% gelatin. The prepared mixture was deposited on a confocal dish and kept at 4°C for 12 min. After adding 200 μL premixed collagen solution to cover the gel aliquots, the dish was put into 37°C incubator immediately for 30 min. Then, 1 mL PBS was added. The fluorescent signal of the gel aliquots was measured every 5 min for the following 3 h on an inverted microscope (Nikon ECLIPSE Ti, Japan), during which PBS was replaced with fresh PBS every 1 hour.

### 4.4 Cavity Structure Characterization

The collagen fiber of the cavity structure was imaged with a reflection model under a Laica confocal fluorescence microscope (Leica TCS SP8, Germany). Excitation wavelength was 488 nm, and the detection wavelength ranged from 478 to 498 nm with an RT15/85 beamsplitter. The 3D image was reconstructed using the 3D viewer/volume viewer plugin in Fiji (an extended distribution of ImageJ) software.

Fluorescent Nile Red Particles with diameter of 0.87 μm (Spherotech, USA) were pre‐mixed with Collagen I preparation solution at 4°C, and other experimental operations were the same as the cavity formation process. The structure was imaged with a Z‐stack capture model with a step size of 4.9 μm under a Laica confocal fluorescence microscope (Leica TCS SP8, Germany). Excitation wavelength was set as 552 nm, and fluorescent signal was collected with the wavelength range from 560 to 660 nm with a PMT detector. The 3D image and corresponding orthogonal view were reconstructed by Fiji.

### 4.5 Cell Morphology Record

After incubation for 6–20 h based on cell types, immunofluorescence analysis was conducted to record cell morphology with the following procedures. Cell medium was aspirated and 4% prewarmed paraformaldehyde (Biorgin, China) was added to fix the cell at RT for 30 min. After that, sterile PBS was used to wash cells for three times and 0.5% (v/v) Triton X‐100 (in PBS, Amresco, USA) was added to permeabilize cells at RT for 10 min. Subsequently, 1 mL TRITC Phalloidin working solution (Solarbio, China) containing 1% BSA (Solarbio, China) was added into the dish and incubated for 30 min at RT in the dark. After washing cells, Hoechst 33342 solution (Thermo Fisher, USA) with 1:500 diluted in PBS was added to stain cell nucleuses at RT for 15 min. Imaged fluorescence pictures under the Laica confocal fluorescence microscope after washing cells. The obtained images were visualized and statisticized by Fiji software.

For quantifying cell morphology, shape descriptor parameters were calculated for at least 60 cells. The obtained raw data were first calibrated by removing outliers based on box plots in SPSS statistics and then plotted in GraphPad Prism or Origin software.

### 4.6 Cell Dynamic Tracking

Single cell dynamic tracking from 3 to 6 h was recorded by an inverted microscope. Abnormal cell movement trajectories resulting from cells overlapping or touching other cells during the tracking process were kicked off manually in Fiji software. The data handling procedure was operated as follows: scale was set as 0.655 μm/pixel; abnormal cell trajectories were removed; Trackmate plug‐in (cell estimated diameter was set as 25 μm; linking max distance was set as 20 μm; gap‐closing max distance was set as 20 μm) was conducted.

### 4.7 Bulk mRNA‐Seq Analysis Operation

For cells in the cavity structure, collagen gel was first removed carefully using 10 μL pipette tip, and naked cells on the glass bottom of the dish were then washed using PBS. Subsequently, 1 mL Collagenase I solution (final concentration of 160 U/mL in PBS) was added to the dish and incubated at 37°C for 30 min. Cells were centrifuged at 100 g for 5 min and then collected. After adding 100 μL PBS, Trypan Blue was used to count the live cell number. Then, cell lysate for transcriptome analysis was added and Smart‐seq2 sequencing was performed (LC‐Bio Technology CO. Ltd., Hangzhou, China).

For cells under hydrogel, collagen gel was first transferred into a new confocal dish using 10 μL pipette tip carefully. The gel was washed using PBS three times. Collagenase I solution was added and incubated with gel at 37°C for 30 min. Then, cells were centrifuged at 100 g for 5 min and treated with the same procedures as cells in cavity group.

For cells on the dish, cells were washed with PBS and then 1 mL Collagenase I solution was added. Following operations were the same as operations in the cavity group.

### 4.8 Bulk RNA‐Seq Data Processing

Raw sequencing data are deposited in the Sequence Read Archive database with accession code: PRJNA1269746. Raw data were first evaluated by FastQC (version 0.12.1). Then, it was treated by Trim galore (version 0.6.10) with the filter of adaptor‐containing reads, low‐quality reads (*Q* < 20), with N content larger than 5% (7 bases for total 150 bases). The obtained clean data were aligned with Reference GRCh38.115 by Hisat 2 (version 2.2.2) and quantified by featureCounts (version 2.1.1) to obtain the raw gene expression count matrix.

All downstream data processing and figure generation were conducted in the R programming environment (version 4.3.3) via RStudio (version 2025.09.0 + 387). A threshold that genes were expressed in at least two samples out of three replicates and the total count should be no less than 10 across all samples was established to filter out lowly expressed genes. Differentially Expressed Genes (DEGs) were selected by the edgeR package (version 4.4.2). For volcano analysis, the standard of FC > 2 & FDR < 0.05 was used for DEGs. GO and KEGG enrichment were conducted by ClusterProfiler package (version 4.10.1) with a relaxed threshold (FC > 1.2, FDR < 0.05) to capture more biological processes. GSEA was performed by GSEABase package (version 1.64.0). Macropinocytosis‐related gene sets were expanded via Protein‐Protein Interaction Networks (PPI) in STRING database based the genes in GOBP_MACROPINOCYTOSIS as seed nodes with active interaction sources of Experiments, Databases, and Co‐expression, minimum required interaction score of high confidence (0.700), and max number of interactors to show first shell of no more than 50 interactors and 2nd shell of no more than 10 interactors.

### 4.9 FITC‐Collagen Diffusion Test

FITC labeled Collagen I with a molecular weight of 0–25 kDa was mixed with normal Collagen I to prepare collagen gel. Final concentrations of FITC‐Collagen I and Collagen I were set as 2 mg/mL for the experiment. The gel preparation and operation were the same as previous procedures to produce cavity structure samples and gel control samples. MDA‐MB‐231 cells were used in the experiment to detect the uptake of FITC‐collagen. Cells were observed under a Laica confocal fluorescent microscope with the following parameters: HCX PLAPO CS 10x/0.40 DRY objective with DD 488/522 beamsplitter; Excitation wavelength 488 nm with signal strength 2%; Emission wavelength 500–580 nm; PMT detector with gain 700; Pinhole 1.00; Zoom factor 10.00.

### 4.10 Inhibition Test

Stock solutions of GLPG 0187 (Aladdin, China), cytochalasin D (Yeasen, China), dynasore (MCE, China) and genistein (MCE, China) were prepared in DMSO (Aladdin, China) based on their solubility and stored at −20°C as stock aliquots. GsMTx4 (MCE, China) stock was prepared by dissolving it in sterile water and stored at −20°C. The influence of 10 μM GLPG 0187, 2 μM GsMTx4, 5 μM cytochalasin D, 100 μM dynasore, and 200 μM genistein (in cell medium) on cell viability and proliferation was tested using the Cell Counting Kit‐8 (CCK8) assay (Solarbio, China). A total of 100 μL cell suspension with the concentration of 5E4 cells/mL was added to a 96‐well plate and incubated for 24 h in the cell culture incubator. Cell medium was replaced with new 100 μL cell medium containing GLPG 0187, GsMTx4, cytochalasin D, dynasore, or genistein. After 3 h, 10 μL CCK8 solution was added to the cell medium and incubated for another 3 h. The absorbance at 450 nm was measured using a Microplate Reader (Thermo Fisher, USA) and showed negligible effects of inhibitors on cells.

The gel mixture of FITC‐Collagen I and Collagen I was first prepared to form the cavity structure. After the solidification of the collagen gel, cell medium containing GLPG 0187, GsMTx4, cytochalasin D, dynasore or genistein was added for another 3 h. To demonstrate the neglectable influence of DMSO, the solvent of the stock solutions of most inhibitors with different DMSO concentration of 0, 0.1%, and 0.2% in DMEM medium (the involved DMSO concentration for all inhibitors) was added into the cavity samples to observe the influence of the FITC‐collagen absorption for cells parallelly at the same time. Then the medium with diffused FITC‐Collagen I was replaced with fresh cell medium, and cells were observed under the Laica confocal fluorescent microscope with the following parameters: HCX IRAPO CS 25x/0.95 WATER objective with DD 488/522 beamsplitter; Excitation wavelength 488 nm with signal strength 0.2%; HyD detector with gain 10%; Emission wavelength 500–580 nm; Pinhole 1.00; Zoom factor 8.00.

### 4.11 Inhibition Verification

For the low‐temperature (4°C) test, cells were incubated at 4°C for 3 h after the gelatinization of the Collagen I and FITC‐collagen mixture. All other steps were identical to the standard cavity detection process. For serum‐deprivation detection, cells were pre‐incubated in serum‐free (basic) medium for 1 h prior to the cell collection. Other experimental operations were the same as the previous cavity detection process except for conducting the test under serum‐free conditions. For the EIPA inhibition test, cells were pre‐treated with 20 μM EIPA for 1 h as well. After pre‐treatment, cells were collected and resuspended at a final density of 4E5 cells/mL. Gels were prepared with a final concentration of 2 mg/mL Collagen I and 0.1 mg/mL FITC‐collagen I. The incubation was performed after the cavity formation in EIPA‐contained medium. For all conditions, the incubation time was fixed at 3 h. Then, cellular uptake of FITC‐collagen I was quantified using a Leica confocal fluorescence microscope equipped with an HCX IRAPO CS 25×/0.95 W water‐immersion objective.

### 4.12 COMSOL Multiphysics

Numerical simulations were performed using COMSOL Multiphysics (version 6.0, COMSOL Inc., USA) to model particle transport toward a porous interface. A two‐dimensional channel geometry (1500 μm in length) was constructed under the assumption of infinite extent in the transverse direction. Fluid flow was described by the Laminar Flow interface, while the culture medium was approximated as water (*ρ* = 1 × 10^3^  kg m^−3^, *μ* = 1 × 10^−3^ Pa s). The ECM was modeled as a porous interface located at the downstream boundary, characterized by an effective permeability (*k* = 1 × 10^−11^ m^2^) and porosity (*ε* = 0.7). Particle transport was modeled using the Transport of Diluted Species in Porous Media interface, incorporating advection, diffusion, and reaction, with a diffusion coefficient D = 1 × 10^−12^ m^2^/s. Particle retention at the porous interface was described using a first‐order sink condition (*R* = −k_r c,k_*r* = 5  s^−1^), representing particle trapping within the ECM. A uniform inlet velocity was applied at the channel entrance, while a prescribed concentration (c_0) was imposed at the porous boundary to represent local particle enrichment. Time‐dependent simulations were performed with locally refined meshing near the interface to resolve concentration gradients. The simulations yielded a steady‐state spatial distribution characterized by pronounced particle accumulation at the porous interface and a monotonic decay of concentration with increasing distance from the interface, forming a long‐range gradient. The reported concentration fields were normalized to the prescribed boundary concentration, such that c^* = c/c_0.

### 4.13 Extracellular Vesicle Size Characterization

Cell medium from gel, cavity, and dish samples was collected and operated following the manufacturer's product instruction of the Extracellular Vesicle Isolation kit (Takara, China) to obtain the vesicle‐contained solution. After dilution of 50 times, the obtained solution was injected into NanoSight NS300 (Malvern Instruments Ltd., UK) for Nanoparticle Tracking Analysis (NTA) analysis with the following experimental parameters: Camera Type, sCMOS; Laser Type, Blue405; Camera Level, 15; Slider Shutter, 1206; Slider gain, 245; FPS, 25.0; Number of Frames, 1498; Number of captures, 3; Capture durations, 60; Continuous syringe pump flow, 100.

### 4.14 Data Statistical Analysis

Data were treated by SPSS statistics software and visualized by GraphPad Prism 8.0 or Origin 2025 software. All quantitative data are presented as means ± SD. Statistical significance of differences between two groups was determined by a two‐tailed Student's t‐test and Bonferroni post‐test (*p* < 0.05 was considered as significant difference: ∗*p* < 0.05, ∗∗*p* < 0.01, ∗∗∗*p* < 0.001).

## Author Contributions

T.C. and F.Y. contributed to the conceptualization and methodology. T.C. conducted the investigation and wrote the original draft. C.Y., Z.W., C.S., Z.X., and B.C. participated in the data analysis and discussion. J.Z., Y.W., and F.Y. supervised the study, with F.Y. also involved in manuscript review.

## Ethics Statement

The authors have nothing to report.

## Conflicts of Interest

Fangfu Ye is an associate editor of *Smart Medicine* and was not involved in the editorial review or the decision to publish this article. All authors declare that there are no competing interests.

## Supporting information


Supporting Information S1

## Data Availability

The data that support the findings of this study are available from the corresponding author upon reasonable request.
