# Supplementary material for: Non‐Contact Transspatial Regulatory Function of the Extracellular Matrix
Source: Smart Med. 2026 Jul 24;5(4):e70046. doi: 10.1002/smmd.70046 (PMC13410397; doi:10.1002/smmd.70046)
Supplement: Supplementary file 1 — Supporting Information S1 [file SMMD-5-e70046-s001.doc]

Supporting Information

Non-contact Transspatial Regulatory Function of the Extracellular Matrix

*Ting Cao,^1,2,3^* Chen Yang,^3^ Zhengyang Wang,^3^ Changmin Shao,^4^ Ziye Xu,^1,2^ Baode Chen,^1^ Jiayu Zhang,^5^* Yongcheng Wang,^1,2^* and Fangfu Ye^3,4^**

1. Department of Laboratory Medicine of The First Affiliated Hospital & Liangzhu Laboratory, Zhejiang University School of Medicine, Hangzhou 310000, China.

2. Zhejiang Key Laboratory of Clinical In Vitro Diagnostic Techniques, Hangzhou 310006, China.

3. Beijing National Laboratory for Condensed Matter Physics and Laboratory of Soft Matter Physics, Institute of Physics, Chinese Academy of Sciences, Beijing 100190, China.

4. Oujiang Laboratory (Zhejiang Lab for Regenerative Medicine, Vision and Brain Health), Wenzhou Institute, University of Chinese Academy of Sciences, Wenzhou 325001, China.

5. School of Traditional Chinese Medicine, Shandong Medical and Pharmaceutical University, Yantai 264003, China.

**Correspondence**: fye@iphy.ac.cn; yongcheng@zju.edu.cn; zhangjiayu0615@bzmc.edu.cn; tingcao@zju.edu.cn

**Figure S1. The image and 3D reconstruction of the cavity structure by collagen fiber under a confocal microscopy with a reflection model.**

**Figure S2.** The picture of cell-in-cavity structure underneath (**A**) 2 mg/mL and (**B**) 4 mg/mL collagen gel.

In some cavities (Figure S2A, 2 mg/mL), few cells have another two statuses that are cells in gel (blue wireframe) and cells adhering on gel (orange wireframe), respectively. This situation can be avoided with the increase of hydrogel stiffness, such as the increase of collagen gel to 4 mg/mL or other biological hydrogels. They almost have negligible influence on the cells in cavity based on other experimental data result in following experiment.

**Figure S3. The distribution of data treated by outlier deficiency method for *Cir*., *AR*, *Rou*., *Sol*., and *ACS*.**

**Figure S4. The neglectable difference of *Cir*., *AR*, *Rou*., *Sol*., and *ACS* results between dish-gelatin and dish-DMEM.**

**Figure S5. Images of partial raw cell moving trajectory for dish, cavity, and gel samples.**

**Figure S6. Quality control analysis before bulk RNAseq for dish, cavity, and gel samples.**

**Figure S7. GSEA enrichment results in Hallmark Gene Sets for DEGs between cavity and dish groups.**

**Figure S8. GSEA enrichment results of (A) GOBP_PINOCYTOSIS, (B) GOBP_CLATHRIN_DEPENDENT_ENDOCYTOSIS, and (C) GOBP_CAVEOLIN_MADIATED_ENDOCYTOSIS for DEGs between cavity and dish groups.**


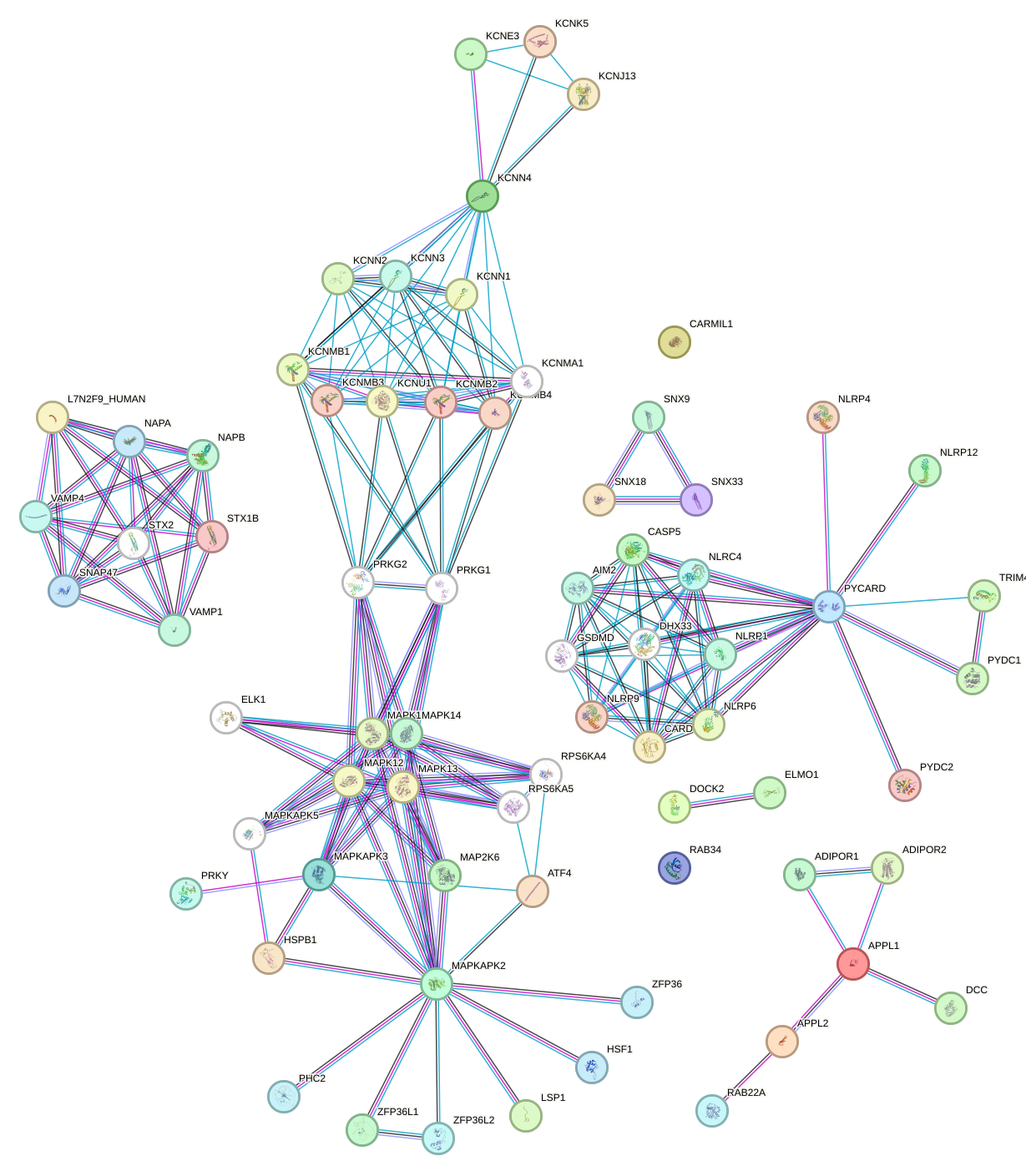


**Figure S9. The expanded macropinocytosis-related gene network by Protein-Protein Interaction in String database.**

**Figure S10. The GO and KEGG enrichment results for the DEGs between gel and cavity samples.**

**Figure S11. The GSEA analysis results: (A) Hallmark gene sets, (B) GOBP_PINOCYTOSIS GOBP_MACROPINOCYTOSIS, and (C) MACROPINOCYTOSIS _complete65 for the DEGs between gel and cavity samples.**

**Figure S13. The GO and KEGG enrichment results for the DEGs between gel and dish samples.**

**Figure S13. The GSEA analysis results (A) Hallmark gene sets and (B) GOBP_CLATHRIN_DEPENDENT_ENDOCYTOSIS for the DEGs between gel and dish samples.**

**Figure S14. The electrophoresis result for FITC-collagen.**

**Figure S15.** **Cell viability and proliferation characterization for different inhibitors by CCK8 assay.**

**Figure S16.** **The influence of DMSO concentration on the uptake of the FITC-collagen (0, 0.1%, and 0.2% are the three kinds of DMSO concentration for the five inhibitors).**

**Figure S17. The dual effect of GsMTx4 on FITC-collagen absorption with some cells inhibited and others not.**

**Figure S18. Vesical bubbles carrying strong green fluorescent signal in GLPG 0187 test.**

**Table S1.** **The difference of data treated by outlier deletion (dark background color) and outlier deficiency (light background color) methods.**

**Table S2.** Enriched GO terms for the comparison between cavity and dish groups.

| **GO-BP** | | | |
| --- | --- | --- | --- |
| **ID** | **Description** | **GeneRatio** | **BgRatio** |
| GO:0042542 | response to hydrogen peroxide | 25/1302 | 101/18870 |
| GO:0042254 | ribosome biogenesis | 51/1302 | 325/18870 |
| GO:0016072 | rRNA metabolic process | 44/1302 | 264/18870 |
| GO:0042273 | ribosomal large subunit biogenesis | 20/1302 | 76/18870 |
| GO:0097193 | intrinsic apoptotic signaling pathway | 48/1302 | 319/18870 |
| GO:0006364 | rRNA processing | 37/1302 | 225/18870 |
| GO:0006979 | response to oxidative stress | 55/1302 | 400/18870 |
| GO:1901873 | regulation of post-translational protein modification | 43/1302 | 284/18870 |
| GO:0006457 | protein folding | 36/1302 | 223/18870 |
| GO:0031647 | regulation of protein stability | 46/1302 | 325/18870 |
| GO:2001233 | regulation of apoptotic signaling pathway | 53/1302 | 398/18870 |
| GO:0000302 | response to reactive oxygen species | 33/1302 | 205/18870 |
| GO:0008380 | RNA splicing | 60/1302 | 478/18870 |
| GO:0043122 | regulation of canonical NF-kappaB signal transduction | 39/1302 | 265/18870 |
| GO:0062197 | cellular response to chemical stress | 44/1302 | 317/18870 |
| GO:0007249 | canonical NF-kappaB signal transduction | 42/1302 | 300/18870 |
| GO:0000470 | maturation of LSU-rRNA | 10/1302 | 28/18870 |
| GO:1903320 | regulation of protein modification by small protein conjugation or removal | 36/1302 | 243/18870 |
| GO:0071243 | cellular response to arsenic-containing substance | 8/1302 | 18/18870 |
| GO:0010506 | regulation of autophagy | 47/1302 | 355/18870 |
| GO:0034599 | cellular response to oxidative stress | 37/1302 | 255/18870 |
| GO:0034976 | response to endoplasmic reticulum stress | 38/1302 | 266/18870 |
| GO:0016032 | viral process | 54/1302 | 432/18870 |
| GO:0048545 | response to steroid hormone | 44/1302 | 330/18870 |
| GO:0009615 | response to virus | 54/1302 | 436/18870 |
| GO:0006986 | response to unfolded protein | 24/1302 | 137/18870 |
| GO:0061684 | chaperone-mediated autophagy | 7/1302 | 15/18870 |
| GO:1901293 | nucleoside phosphate biosynthetic process | 40/1302 | 297/18870 |
| GO:0046685 | response to arsenic-containing substance | 10/1302 | 32/18870 |
| GO:0070301 | cellular response to hydrogen peroxide | 15/1302 | 67/18870 |
| GO:0072594 | establishment of protein localization to organelle | 55/1302 | 459/18870 |
| GO:2001242 | regulation of intrinsic apoptotic signaling pathway | 29/1302 | 190/18870 |
| GO:0006268 | DNA unwinding involved in DNA replication | 8/1302 | 21/18870 |
| GO:0000463 | maturation of LSU-rRNA from tricistronic rRNA transcript (SSU-rRNA, 5.8S rRNA, LSU-rRNA) | 7/1302 | 16/18870 |
| GO:0034470 | ncRNA processing | 53/1302 | 439/18870 |
| GO:0009142 | nucleoside triphosphate biosynthetic process | 22/1302 | 127/18870 |
| GO:0071496 | cellular response to external stimulus | 44/1302 | 346/18870 |
| GO:0009165 | nucleotide biosynthetic process | 39/1302 | 295/18870 |
| GO:0001893 | maternal placenta development | 10/1302 | 35/18870 |
| GO:0035966 | response to topologically incorrect protein | 25/1302 | 159/18870 |
| GO:1901875 | positive regulation of post-translational protein modification | 25/1302 | 159/18870 |
| GO:0006260 | DNA replication | 37/1302 | 278/18870 |
| GO:0009260 | ribonucleotide biosynthetic process | 32/1302 | 228/18870 |
| GO:0019058 | viral life cycle | 41/1302 | 321/18870 |
| GO:0031668 | cellular response to extracellular stimulus | 37/1302 | 281/18870 |
| GO:1901798 | positive regulation of signal transduction by p53 class mediator | 9/1302 | 30/18870 |
| GO:0071826 | protein-RNA complex organization | 33/1302 | 243/18870 |
| GO:0046686 | response to cadmium ion | 13/1302 | 59/18870 |
| GO:0002181 | cytoplasmic translation | 24/1302 | 156/18870 |
| GO:0046390 | ribose phosphate biosynthetic process | 32/1302 | 235/18870 |
| GO:0009201 | ribonucleoside triphosphate biosynthetic process | 20/1302 | 119/18870 |
| GO:1902229 | regulation of intrinsic apoptotic signaling pathway in response to DNA damage | 10/1302 | 38/18870 |
| GO:0046697 | decidualization | 8/1302 | 25/18870 |
| GO:0033157 | regulation of intracellular protein transport | 31/1302 | 226/18870 |
| GO:0008630 | intrinsic apoptotic signaling pathway in response to DNA damage | 18/1302 | 102/18870 |
| GO:0043620 | regulation of DNA-templated transcription in response to stress | 10/1302 | 39/18870 |
| GO:0009267 | cellular response to starvation | 26/1302 | 179/18870 |
| GO:0007059 | chromosome segregation | 49/1302 | 424/18870 |
| GO:0031669 | cellular response to nutrient levels | 33/1302 | 251/18870 |
| GO:0043161 | proteasome-mediated ubiquitin-dependent protein catabolic process | 52/1302 | 458/18870 |
| GO:0006261 | DNA-templated DNA replication | 24/1302 | 161/18870 |
| GO:0000492 | box C/D snoRNP assembly | 5/1302 | 10/18870 |
| GO:0043618 | regulation of transcription from RNA polymerase II promoter in response to stress | 9/1302 | 33/18870 |
| GO:0009411 | response to UV | 23/1302 | 152/18870 |
| GO:0030174 | regulation of DNA-templated DNA replication initiation | 6/1302 | 15/18870 |
| GO:0048308 | organelle inheritance | 6/1302 | 15/18870 |
| GO:0048313 | Golgi inheritance | 6/1302 | 15/18870 |
| GO:0045862 | positive regulation of proteolysis | 42/1302 | 350/18870 |
| GO:0002262 | myeloid cell homeostasis | 25/1302 | 173/18870 |
| GO:0034614 | cellular response to reactive oxygen species | 23/1302 | 154/18870 |
| GO:0022618 | protein-RNA complex assembly | 31/1302 | 235/18870 |
| GO:0070059 | intrinsic apoptotic signaling pathway in response to endoplasmic reticulum stress | 13/1302 | 65/18870 |
| GO:0043123 | positive regulation of canonical NF-kappaB signal transduction | 27/1302 | 196/18870 |
| GO:0046822 | regulation of nucleocytoplasmic transport | 18/1302 | 109/18870 |
| GO:0006750 | glutathione biosynthetic process | 6/1302 | 16/18870 |
| GO:0050821 | protein stabilization | 29/1302 | 217/18870 |
| GO:0061687 | detoxification of inorganic compound | 7/1302 | 22/18870 |
| GO:0042594 | response to starvation | 29/1302 | 218/18870 |
| GO:0071383 | cellular response to steroid hormone stimulus | 28/1302 | 208/18870 |
| GO:0072332 | intrinsic apoptotic signaling pathway by p53 class mediator | 15/1302 | 84/18870 |
| GO:0010595 | positive regulation of endothelial cell migration | 20/1302 | 130/18870 |
| GO:0051235 | maintenance of location | 41/1302 | 350/18870 |
| GO:0032392 | DNA geometric change | 15/1302 | 85/18870 |
| GO:0050691 | regulation of defense response to virus by host | 10/1302 | 44/18870 |
| GO:2001234 | negative regulation of apoptotic signaling pathway | 31/1302 | 243/18870 |
| GO:0006913 | nucleocytoplasmic transport | 39/1302 | 330/18870 |
| GO:0051169 | nuclear transport | 39/1302 | 330/18870 |
| GO:1903322 | positive regulation of protein modification by small protein conjugation or removal | 20/1302 | 132/18870 |
| GO:0006270 | DNA replication initiation | 9/1302 | 37/18870 |
| GO:0071276 | cellular response to cadmium ion | 9/1302 | 37/18870 |
| GO:1902230 | negative regulation of intrinsic apoptotic signaling pathway in response to DNA damage | 8/1302 | 30/18870 |
| GO:0009152 | purine ribonucleotide biosynthetic process | 28/1302 | 213/18870 |
| GO:0009145 | purine nucleoside triphosphate biosynthetic process | 18/1302 | 114/18870 |
| GO:0034504 | protein localization to nucleus | 37/1302 | 310/18870 |
| GO:0000491 | small nucleolar ribonucleoprotein complex assembly | 5/1302 | 12/18870 |
| GO:0000727 | double-strand break repair via break-induced replication | 5/1302 | 12/18870 |
| GO:0006534 | cysteine metabolic process | 5/1302 | 12/18870 |
| GO:0016050 | vesicle organization | 42/1302 | 366/18870 |
| GO:2001238 | positive regulation of extrinsic apoptotic signaling pathway | 11/1302 | 53/18870 |
| GO:0009141 | nucleoside triphosphate metabolic process | 25/1302 | 184/18870 |
| GO:0006164 | purine nucleotide biosynthetic process | 32/1302 | 258/18870 |
| GO:0032508 | DNA duplex unwinding | 14/1302 | 79/18870 |
| GO:0050688 | regulation of defense response to virus | 16/1302 | 97/18870 |
| GO:0006783 | heme biosynthetic process | 8/1302 | 31/18870 |
| GO:0019184 | nonribosomal peptide biosynthetic process | 6/1302 | 18/18870 |
| GO:0072331 | signal transduction by p53 class mediator | 24/1302 | 175/18870 |
| GO:2001235 | positive regulation of apoptotic signaling pathway | 21/1302 | 145/18870 |
| GO:0042168 | heme metabolic process | 10/1302 | 46/18870 |
| **GO-CC** | | | |
| **ID** | **Description** | **GeneRatio** | **BgRatio** |
| GO:0005925 | focal adhesion | 63/1329 | 421/19886 |
| GO:0030055 | cell-substrate junction | 64/1329 | 431/19886 |
| GO:0042470 | melanosome | 25/1329 | 112/19886 |
| GO:0048770 | pigment granule | 25/1329 | 112/19886 |
| GO:0031983 | vesicle lumen | 46/1329 | 326/19886 |
| GO:0034774 | secretory granule lumen | 45/1329 | 322/19886 |
| GO:0060205 | cytoplasmic vesicle lumen | 45/1329 | 325/19886 |
| GO:0005759 | mitochondrial matrix | 60/1329 | 487/19886 |
| GO:0005819 | spindle | 54/1329 | 431/19886 |
| GO:0005635 | nuclear envelope | 59/1329 | 494/19886 |
| GO:1904813 | ficolin-1-rich granule lumen | 22/1329 | 124/19886 |
| GO:0005743 | mitochondrial inner membrane | 58/1329 | 497/19886 |
| GO:0098687 | chromosomal region | 49/1329 | 399/19886 |
| GO:0140534 | endoplasmic reticulum protein-containing complex | 22/1329 | 126/19886 |
| GO:0072686 | mitotic spindle | 28/1329 | 184/19886 |
| GO:1905368 | peptidase complex | 21/1329 | 120/19886 |
| GO:0031968 | organelle outer membrane | 34/1329 | 246/19886 |
| GO:0005765 | lysosomal membrane | 52/1329 | 441/19886 |
| GO:0098852 | lytic vacuole membrane | 52/1329 | 441/19886 |
| GO:0019867 | outer membrane | 34/1329 | 248/19886 |
| GO:0000228 | nuclear chromosome | 32/1329 | 228/19886 |
| GO:0005770 | late endosome | 40/1329 | 315/19886 |
| GO:0098798 | mitochondrial protein-containing complex | 38/1329 | 300/19886 |
| GO:0031965 | nuclear membrane | 39/1329 | 311/19886 |
| GO:0000502 | proteasome complex | 13/1329 | 60/19886 |
| GO:0022625 | cytosolic large ribosomal subunit | 13/1329 | 60/19886 |
| GO:0044391 | ribosomal subunit | 27/1329 | 188/19886 |
| GO:0005774 | vacuolar membrane | 54/1329 | 484/19886 |
| GO:0000776 | kinetochore | 25/1329 | 171/19886 |
| GO:0101002 | ficolin-1-rich granule | 26/1329 | 185/19886 |
| GO:0005766 | primary lysosome | 23/1329 | 155/19886 |
| GO:0042582 | azurophil granule | 23/1329 | 155/19886 |
| GO:0015934 | large ribosomal subunit | 19/1329 | 117/19886 |
| GO:0005876 | spindle microtubule | 15/1329 | 81/19886 |
| GO:0022626 | cytosolic ribosome | 19/1329 | 118/19886 |
| GO:0030684 | preribosome | 18/1329 | 109/19886 |
| GO:0000118 | histone deacetylase complex | 15/1329 | 82/19886 |
| GO:0101031 | protein folding chaperone complex | 10/1329 | 42/19886 |
| GO:0090575 | RNA polymerase II transcription regulator complex | 32/1329 | 254/19886 |
| GO:0071162 | CMG complex | 5/1329 | 11/19886 |
| GO:0000779 | condensed chromosome, centromeric region | 25/1329 | 182/19886 |
| GO:0005681 | spliceosomal complex | 27/1329 | 205/19886 |
| GO:0005741 | mitochondrial outer membrane | 28/1329 | 217/19886 |
| GO:0001725 | stress fiber | 13/1329 | 70/19886 |
| GO:0097517 | contractile actin filament bundle | 13/1329 | 70/19886 |
| GO:0005643 | nuclear pore | 16/1329 | 97/19886 |
| GO:0032432 | actin filament bundle | 14/1329 | 79/19886 |
| GO:0001650 | fibrillar center | 21/1329 | 150/19886 |
| GO:0005840 | ribosome | 30/1329 | 246/19886 |
| GO:0000812 | Swr1 complex | 5/1329 | 13/19886 |
| GO:0031261 | DNA replication preinitiation complex | 5/1329 | 13/19886 |
| GO:0030687 | preribosome, large subunit precursor | 6/1329 | 19/19886 |
| GO:0042641 | actomyosin | 13/1329 | 76/19886 |
| GO:0099522 | cytosolic region | 6/1329 | 20/19886 |
| GO:1990498 | mitotic spindle microtubule | 5/1329 | 14/19886 |
| GO:1904949 | ATPase complex | 20/1329 | 146/19886 |
| GO:0098800 | inner mitochondrial membrane protein complex | 21/1329 | 158/19886 |
| GO:0000151 | ubiquitin ligase complex | 35/1329 | 314/19886 |
| GO:0035770 | ribonucleoprotein granule | 31/1329 | 270/19886 |
| GO:0042581 | specific granule | 21/1329 | 160/19886 |
| GO:0061695 | transferase complex, transferring phosphorus-containing groups | 34/1329 | 306/19886 |
| GO:0070469 | respirasome | 15/1329 | 102/19886 |
| GO:0031902 | late endosome membrane | 22/1329 | 175/19886 |
| GO:1904724 | tertiary granule lumen | 10/1329 | 55/19886 |
| GO:0005788 | endoplasmic reticulum lumen | 34/1329 | 313/19886 |
| GO:1905369 | endopeptidase complex | 13/1329 | 84/19886 |
| GO:0008180 | COP9 signalosome | 5/1329 | 17/19886 |
| GO:0000793 | condensed chromosome | 31/1329 | 281/19886 |
| GO:0000775 | chromosome, centromeric region | 29/1329 | 258/19886 |
| GO:0000781 | chromosome, telomeric region | 21/1329 | 171/19886 |
| GO:0016469 | proton-transporting two-sector ATPase complex | 9/1329 | 50/19886 |
| **GO-MF** | | | |
| **ID** | **Description** | **GeneRatio** | **BgRatio** |
| GO:0045296 | cadherin binding | 50/1309 | 334/18496 |
| GO:0061629 | RNA polymerase II-specific DNA-binding transcription factor binding | 51/1309 | 344/18496 |
| GO:0016922 | nuclear receptor binding | 26/1309 | 137/18496 |
| GO:0140297 | DNA-binding transcription factor binding | 61/1309 | 477/18496 |
| GO:0031625 | ubiquitin protein ligase binding | 41/1309 | 308/18496 |
| GO:0051082 | unfolded protein binding | 21/1309 | 122/18496 |
| GO:0044389 | ubiquitin-like protein ligase binding | 42/1309 | 327/18496 |
| GO:0051087 | protein-folding chaperone binding | 22/1309 | 133/18496 |
| GO:0000979 | RNA polymerase II core promoter sequence-specific DNA binding | 7/1309 | 19/18496 |
| GO:0003697 | single-stranded DNA binding | 20/1309 | 118/18496 |
| GO:0016765 | transferase activity, transferring alkyl or aryl (other than methyl) groups | 13/1309 | 60/18496 |
| GO:1990381 | ubiquitin-specific protease binding | 7/1309 | 21/18496 |
| GO:0003735 | structural constituent of ribosome | 25/1309 | 176/18496 |
| GO:0070325 | lipoprotein particle receptor binding | 8/1309 | 29/18496 |

**Table S3.** Enriched KEGG terms for the comparison between cavity and dish groups.

| **ID** | **category** | **subcategory** | **Description** | **GeneRatio** | **BgRatio** |
| --- | --- | --- | --- | --- | --- |
| hsa04141 | Genetic Information Processing | Folding, sorting and degradation | Protein processing in endoplasmic reticulum | 31/688 | 174/9375 |
| hsa05016 | Human Diseases | Neurodegenerative disease | Huntington disease | 45/688 | 308/9375 |
| hsa05012 | Human Diseases | Neurodegenerative disease | Parkinson disease | 40/688 | 268/9375 |
| hsa05222 | Human Diseases | Cancer: specific types | Small cell lung cancer | 19/688 | 93/9375 |
| hsa05134 | Human Diseases | Infectious disease: bacterial | Legionellosis | 14/688 | 56/9375 |
| hsa05131 | Human Diseases | Infectious disease: bacterial | Shigellosis | 37/688 | 253/9375 |
| hsa04668 | Environmental Information Processing | Signal transduction | TNF signaling pathway | 22/688 | 119/9375 |
| hsa01240 | Metabolism | Global and overview maps | Biosynthesis of cofactors | 26/688 | 154/9375 |
| hsa05146 | Human Diseases | Infectious disease: parasitic | Amoebiasis | 19/688 | 103/9375 |
| hsa05120 | Human Diseases | Infectious disease: bacterial | Epithelial cell signaling in Helicobacter pylori infection | 15/688 | 71/9375 |
| hsa05022 | Human Diseases | Neurodegenerative disease | Pathways of neurodegeneration - multiple diseases | 57/688 | 480/9375 |
| hsa03030 | Genetic Information Processing | Replication and repair | DNA replication | 10/688 | 36/9375 |
| hsa03060 | Genetic Information Processing | Folding, sorting and degradation | Protein export | 9/688 | 31/9375 |
| hsa04210 | Cellular Processes | Cell growth and death | Apoptosis | 22/688 | 137/9375 |
| hsa04657 | Organismal Systems | Immune system | IL-17 signaling pathway | 17/688 | 94/9375 |
| hsa05020 | Human Diseases | Neurodegenerative disease | Prion disease | 36/688 | 275/9375 |
| hsa05167 | Human Diseases | Infectious disease: viral | Kaposi sarcoma-associated herpesvirus infection | 28/688 | 196/9375 |
| hsa05010 | Human Diseases | Neurodegenerative disease | Alzheimer disease | 46/688 | 388/9375 |
| hsa04510 | Cellular Processes | Cellular community - eukaryotes | Focal adhesion | 28/688 | 203/9375 |
| hsa00190 | Metabolism | Energy metabolism | Oxidative phosphorylation | 21/688 | 138/9375 |
| hsa05014 | Human Diseases | Neurodegenerative disease | Amyotrophic lateral sclerosis | 43/688 | 368/9375 |
| hsa03050 | Genetic Information Processing | Folding, sorting and degradation | Proteasome | 10/688 | 46/9375 |
| hsa05170 | Human Diseases | Infectious disease: viral | Human immunodeficiency virus 1 infection | 28/688 | 214/9375 |
| hsa05417 | Human Diseases | Cardiovascular disease | Lipid and atherosclerosis | 28/688 | 216/9375 |
| hsa04621 | Organismal Systems | Immune system | NOD-like receptor signaling pathway | 25/688 | 187/9375 |
| hsa04110 | Cellular Processes | Cell growth and death | Cell cycle | 22/688 | 158/9375 |
| hsa04710 | Organismal Systems | Environmental adaptation | Circadian rhythm | 8/688 | 34/9375 |
| hsa04520 | Cellular Processes | Cellular community - eukaryotes | Adherens junction | 15/688 | 93/9375 |
| hsa04120 | Genetic Information Processing | Folding, sorting and degradation | Ubiquitin mediated proteolysis | 20/688 | 142/9375 |
| hsa05323 | Human Diseases | Immune disease | Rheumatoid arthritis | 15/688 | 95/9375 |
| hsa04064 | Environmental Information Processing | Signal transduction | NF-kappa B signaling pathway | 16/688 | 105/9375 |
| hsa04137 | Cellular Processes | Transport and catabolism | Mitophagy - animal | 16/688 | 105/9375 |
| hsa05163 | Human Diseases | Infectious disease: viral | Human cytomegalovirus infection | 28/688 | 227/9375 |
| hsa05171 | Human Diseases | Infectious disease: viral | Coronavirus disease - COVID-19 | 29/688 | 238/9375 |
